# Short-Term Environmental Stimulation Spatiotemporally Modulates Specific Serotonin Receptor Gene Expression and Behavioral Pharmacology in a Sexually Dimorphic Manner in Huntington’s Disease Transgenic Mice

**DOI:** 10.3389/fnmol.2018.00433

**Published:** 2018-12-10

**Authors:** Michelle S. Zajac, Thibault Renoir, Victoria M. Perreau, Shanshan Li, Wendy Adams, Maarten van den Buuse, Anthony J. Hannan

**Affiliations:** ^1^Florey Institute of Neuroscience and Mental Health, Melbourne Brain Centre, University of Melbourne, Parkville, VIC, Australia; ^2^Department of Anatomy and Neuroscience, University of Melbourne, Parkville, VIC, Australia; ^3^Department of Psychology, University of British Columbia, Vancouver, BC, Canada; ^4^School of Psychology and Public Health, La Trobe University, Melbourne, VIC, Australia

**Keywords:** tandem repeat disorder, polyglutamine disease, neurodegeneration, serotonin, dementia, depression, environmental enrichment, exercise

## Abstract

Huntington’s disease (HD) is a neurodegenerative disorder caused by a tandem repeat mutation encoding an expanded polyglutamine tract in the huntingtin protein, which leads to cognitive, psychiatric and motor dysfunction. Exposure to environmental enrichment (EE), which enhances levels of cognitive stimulation and physical activity, has therapeutic effects on cognitive, affective and motor function of transgenic HD mice. The present study investigated gene expression changes and behavioral pharmacology in male and female R6/1 transgenic HD mice at an early time-point in HD progression associated with onset of cognitive and affective abnormalities, following EE and exercise (wheel running) interventions. We have demonstrated changes in expression levels of the serotonin (5-HT) receptor Htr1a, Htr1b, Htr2a and Htr2c genes (encoding the 5-HT_1A_, 5-HT_1B_, 5-HT_2A_ and 5-HT_2C_ receptors, respectively) in HD brains at 8 weeks of age, using quantitative real-time PCR. In contrast, expression of the serotonin transporter (SerT, also known as 5-HTT or Slc6a4) was not altered in these brains. Furthermore, we identified region-specific, sex-specific and environmentally regulated (comparing EE, exercise and standard housing conditions) impacts on gene expression of particular 5-HT receptors, as well as SerT. For example, SerT gene expression was upregulated by exercise (wheel running from 6 to 8 weeks of age) in the hippocampus. Interestingly, when EE was introduced from 6 to 8 weeks of age, Htr2a gene expression was upregulated in the cortex, striatum and hippocampus of male mice. EE also rescued the functional activity of 5-HT_2_ receptors as observed in the head-twitch test, reflecting sexually dimorphic effects of environmental stimulation. These findings demonstrate that disruption of the serotonergic system occurs early in HD pathogenesis and, together with previous findings, show that the timing and duration of environmental interventions are critical in terms of their ability to modify gene expression. This study is the first to show that EE is able to selectively enhance both gene expression of a neurotransmitter receptor and the functional consequences on behavioral pharmacology, and links this molecular modulation to the therapeutic effects of environmental stimulation in this neurodegenerative disease.

## Introduction

Huntington’s disease (HD) is a neurodegenerative disorder characterized by a triad of symptoms: cognitive deficits culminating in dementia, psychiatric symptoms such as depression and motor dysfunction including uncontrolled movements (chorea). Cognitive deficiencies and psychiatric symptoms begin prior to clinical diagnosis (motor onset) of HD (Zimbelman et al., 2007; Tyebji and Hannan, 2017). The brains of patients diagnosed with HD can display little or no detectable pathological change post-mortem (Vonsattel et al., 1985; Mo et al., 2015). Therefore, understanding subtle molecular changes that occur before clinical onset is very important, and may identify novel therapeutic targets. Transcriptional dysregulation is one of the characteristics of HD pathogenesis and is a possible pathological mechanism of action within the HD brain. Reversing abnormal gene expression may prevent downstream changes leading to neuronal dysfunction. Therefore, identifying early changes in gene expression is an important step in understanding HD pathogenesis and identifying potential therapeutic targets. It is these early changes that need to be targeted with therapies if HD progression is to be stopped or delayed.

It is known that environmental factors affect the time of onset, rate of progression and severity of HD (Sudarsky et al., 1983; Georgiou et al., 1999; Wexler et al., 2004; Friedman et al., 2005; Gomez-Esteban et al., 2007; Trembath et al., 2010). However, human studies have been unable to distinguish which facets of the environment influence disease onset. Therefore, in order to investigate environmental modifiers in HD, animal models must be used. Environmental enrichment (EE) provides increased cognitive, sensory and motor stimulation for experimental animals. EE and voluntary wheel running also affect transcription of specific genes in the cortex and hippocampus of wild-type (WT) rodents (Olsson et al., 1994, 1995; Torasdotter et al., 1996; Rasmuson et al., 1998; Rampon et al., 2000; Pinaud et al., 2001, 2002; Molteni et al., 2002; Mlynarik et al., 2004). Experimentally-enriched environments produce beneficial outcomes for HD mice (Chapillon et al., 1999; van Dellen et al., 2000; van Praag et al., 2000; Hockly et al., 2002; reviewed in Nithianantharajah and Hannan, 2006). Initially, EE was shown to delay onset of motor symptoms in R6/1 HD mice (van Dellen et al., 2000) and this has been extended to other models and phenotypic outcomes (Hockly et al., 2002; Spires et al., 2004; Nithianantharajah et al., 2008; Pang et al., 2009), as well as the beneficial effects of increased voluntary physical activity on running wheels (RWs) (Pang et al., 2006; van Dellen et al., 2008). However, these studies were predominantly performed in later stages of disease progression.

We therefore looked for changes in gene expression in the R6/1 transgenic mouse model of HD at early stages of pathogenesis (8 weeks of age) and also investigated the effects of EE and exercise (wheel running) on these HD mice and WT controls. Our prior findings (Renoir et al., 2011, 2012, 2013; Du et al., 2012). Suggested that serotonergic dysfunction was occurring in R6/1 HD mice. Therefore, in the present study, candidates within the serotonergic system were chosen for investigation, revealing dysregulation in early stages of HD pathogenesis, and differential modulation by environmental interventions.

## Materials and Methods

### Animals and Housing

R6/1 hemizygote males (Mangiarini et al., 1996) were obtained from the Jackson Laboratory (Bar Harbor, ME, United States) and bred with CBB6 (CBAxC57/B6) F1 females to establish an R6/1 colony at the Florey Institute. These mice closely model HD and live for more than 6 months. Genotypes were determined by PCR (Mangiarini et al., 1996) with genomic DNA obtained from toe clips and mice were weaned at 3.5 weeks of age.

The WT and HD male and female mice were separately divided at 6 weeks of age into three groups—standard housed (SH), RW and EE. SH mice were housed in standard mouse boxes (10 cm × 16 cm × 38 cm) containing only bedding, with four mice per box. RW and EE mice were housed in larger sized (15 cm × 28 cm × 38 cm) rat boxes with elevated lids, containing bedding, four mice per box. RW mice were provided with two RWs per box. Housing boxes for EE mice contained a variety of novel objects (e.g., cardboard rolls, wire, mesh, various types of paper, wooden and plastic objects). The objects were changed twice weekly. Additionally, all mice in the EE housing groups were placed in larger (44 cm × 40 cm × 62 cm) activity boxes for 1 h three times weekly. Activity boxes were built up anew on each occasion with novel objects made of plastic, foam, rubber, wood, rope, wire, chains and paper. The differential EE, RW and SH housing conditions were maintained from 6 to 8 weeks of age (i.e., the environmental intervention was performed over 2 weeks).

Mice were housed under a 12/12 light/dark cycle and food and water were provided *ad libitum*. Experiments were approved by the Howard Florey Institute Animal Ethics Committee and followed the guidelines of the National Health and Medical Research Council of Australia.

### Tissue Collection, Dissections and Storage

Mice were killed by cervical dislocation between 9 and 11:30 am over 3 days. Differently housed groups were randomly allocated into balanced batches to eliminate batch effects. For quantitative real-time PCR analysis of gene expression, brains were bisected and hippocampus, striatum and whole cortex dissected out. Samples were immediately frozen on dry ice and transferred to a -80°C freezer for storage until required.

### RNA Extraction, DNase Clean-Up and Analysis

Total RNA from the hippocampus and striatum was extracted using RNeasy Mini kits (Qiagen, Melbourne, VIC, Australia). Total RNA from whole cortex was extracted using RNeasy Midi kits (Qiagen, Melbourne, VIC, Australia). On-column DNase cleanup was performed on all samples using RNase-Free DNase set (Qiagen, Melbourne, VIC, Australia). The concentration and integrity of the extracted total RNA present in the final eluates were determined using an Agilent Bioanalyser 2100 (service provided by Australian Genomics Research Facility, Parkville, VIC, Australia).

### Reverse Transcription

For each RNA sample, 1 μg of total RNA was reverse transcribed into cDNA using GeneAmp RT PCR kit (Applied Biosystems, Foster City, CA, United States) with random hexamers. The reverse transcription reactions were performed on a GeneAmp PCR system (Model 2700, Applied Biosystems, Foster City, CA, United States) at 25°C for 10 min, 48°C for 30 min and 95°C for 5 min. The cDNA products were stored at -20°C for subsequent use.

### Quantitative Real-Time PCR

Expression levels of candidate genes were determined with quantitative real-time PCR performed on the PE-ABI Prism 7700 Sequence detection system version 1.9.1 (Applied Biosystems, Foster City, CA, United States) using SYBR Green JumpStart Taq ReadyMix (Sigma, Saint Louis, MI, United States). Primer 3 software (Rozen and Skaletsky, 2000) or Primer Express Software (Applied Biosystems, Foster City, CA, United States) were used to design reaction primers (Sigma Genosys, Castle Hill, NSW, Australia), which were designed across exon–exon boundaries. The primer sequences used were:

SerT_F:5′-CTTCAGCCCCGGATGGTT-3′;

SerT_R:5′-GTGGACTCATCAAAAAACTGCAAA-3′;

Ht1a_F:5′-CCCCAACGAGTGCACCAT-3′;

Ht1a_R: 5′-GCGCCGAAAGTGGAGTAGAT-3′;

Ht1b_F:5′-CACCAACCTCTCCCACAACT-3′;

Ht1b_R:5′-CCAGAGAGGCGATCAGGTAG-3′;

Ht2a_F:5′-CACTGTGAAGCGAGGCATAA-3′;

Ht2a_R:5′-AAGCCGGAAGTTGTAGCAGA-3′;

Ht2c_F:5′-TGCCATCGTTTGGGCAATA-3′;

Ht2c_R:5′-CGTCCCTCAGTCCAATCACA-3′;

Cyclophilin forward: 5′-CCCACCGTGTTCTTCGACA-3′;

Cyclophilin reverse: 5′-CCAGTGCTCAGAGCTCGAAA-3′.

To determine the optimal working volumes of forward and reverse primers, a set of primer dilutions was conducted in which different combinations of each primer ranging between 0.5 and 3 were used. The combination that yielded the lowest C*t*-value was used as the optimal working volume (data not shown). Optimization of primer efficiencies was carried out prior to commencement of quantification experiments. The real-time PCR cycling conditions were: 50°C for 2 min, 95°C for 10 min, followed by 40 cycles of 95°C for 15 s and 60°C for 1 min. Each sample was processed in triplicate and melt curve analysis was performed on all samples. As a validated endogenous control, cyclophilin A was amplified in separate triplicate reactions for normalization.

Determination of the relative gene expression was performed using the 2^-ΔΔC^*^t^* method (Livak and Schmittgen, 2001). Briefly, the C*t* values (threshold cycle at which the fluorescence intensity exceeds 10× the SD of background fluorescence) of the experimental genes and the control were determined for each sample. The difference in the C*t* values of the mean of the experimental gene triplicates and the mean of the control triplicates was determined (ΔC*t*) for each mouse, normalizing for amount of cDNA in each reaction. The mean ΔC*t* for the WT SH group was calculated, to use as a calibrator, and subtracted from the ΔC*t* of all the mice and giving the ΔΔC*t*. Fold change was then determined by the formula 2^-ΔΔC^*^t^*.

### DOI-Induced Head-Twitches

Eight-week-old mice were injected with the 5-HT_2A_ receptor agonist, 1-(2,5-dimethoxy-4-odophenyl)-2-aminopropane [(±)DOI] (1 mg/kg, i.p.) and immediately placed inside an observation area. The number of head-twitches the mice performed from 15 to 30 min post-injection was manually recorded (Renoir et al., 2011).

### Statistical Analysis

Statistical analysis of quantitative real-time PCR and behavioral pharmacology (DOI) data was performed with the SPSS Package, version 16 (SPSS, Chicago, IL, United States). Fold changes were analyzed by three-way ANOVA (sex × genotype × housing condition). The critical value for significance was set at *p* < 0.05. In both cases, *post hoc* analyses were performed where appropriate using Tukey’s test for housing condition and pairwise testing was conducted when significant interactions were present, using Bonferroni’s adjustment for multiple comparisons. Fold changes were converted into percentage values for graphical representation.

## Results

We used quantitative real-time PCR to compare the effects of EE and RW, relative to standard housing (SH), on serotonergic gene expression in brains of male and female HD mice, and their WT control littermates. The use of these three housing conditions allowed us to compare the relative effects of both cognitive stimulation and physical activity (via EE), vs physical exercise alone (RW). We also investigated the effects of the HD genotype on gene expression, as well as the effects of sex.

### Serotonin Transporter (Slc6a4/SerT) Gene Expression Is Affected by Sex and Environment

**FIGURE 1:**
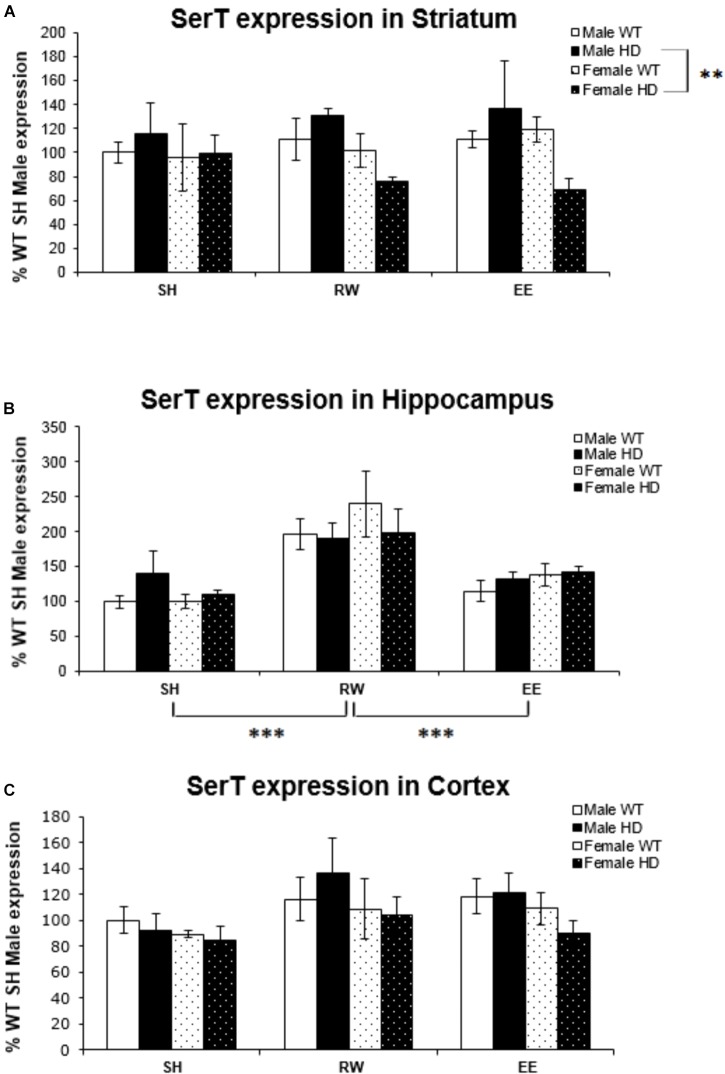
Effects of environmental interventions and sex on SerT mRNA levels in 8-week-old WT and HD mice. **(A)** In Striatum, SerT expression was unaffected by genotype (*p* = 0.862) and housing environment (*p* = 0.867). Sex did significantly affect SerT mRNA levels (*p* = 0.019). There was a significant interaction between genotype × sex (*p* = 0.030); pairwise comparisons showed a significantly less SerT mRNA in female HD mice when compared to male HD mice (*p* = 0.002). **(B)** In Hippocampus, SerT expression was unaffected by genotype (*p* = 0.774) or sex (*p* = 0.506). Housing environment significantly affected SerT expression levels (*p* < 0.001). *Post hoc* testing showed that wheel running (*p* < 0.001), but not environmental enrichment (*p* = 0.510), significantly increased levels of SerT mRNA. **(C)** In Cortex, SerT expression was unaffected by genotype (*p* = 0.816). There were no effects of sex (*p* = 0.065) or housing environment (*p* = 0.063). *n* = 4–6 per group. Results are represented as mean ± SEM. Pairwise comparisons were conducted using Bonferonni’s adjustment for multiple comparisons: ^∗∗^*p* ≤ 0.01; ^∗∗∗^*p* ≤ 0.001.

SerT gene expression was not affected by the HD genotype in the striatum [Figure 1A; *F*(1,67) = 0.030, *p* = 0.862], hippocampus [Figure 1B; *F*(1,68) = 0.083, *p* = 0.774], or cortex [Figure 1C; *F*(1,71) = 0.54, *p* = 0.816]. There was no effect of housing environment in the striatum [Figure 1A; *F*(1,67) = 0.144, *p* = 0.867]. However, there was a significant interaction between genotype × sex [Figure 1A; *F*(1,67) = 4.956, *p* = 0.030]; pairwise comparison showed a significant difference in SerT gene expression levels between male and female HD mice (*p* = 0.002).

Housing environment significantly affected SerT gene expression in the hippocampus [Figure 1B; *F*(2,68) = 17.739, *p* < 0.001]; *post hoc* testing revealed that exposure to wheel running significantly increased SerT mRNA levels when compared to SH mice (*p* < 0.001) and EE mice (*p* < 0.001). In the cortex, there was no effect of housing environment [Figure 1B; *F*(1,71) = 2.863, *p* = 0.065].

### Deficits in Htr1a Gene Expression in HD Mice Are Differentially Modulated by Sex and Environment

**FIGURE 2:**
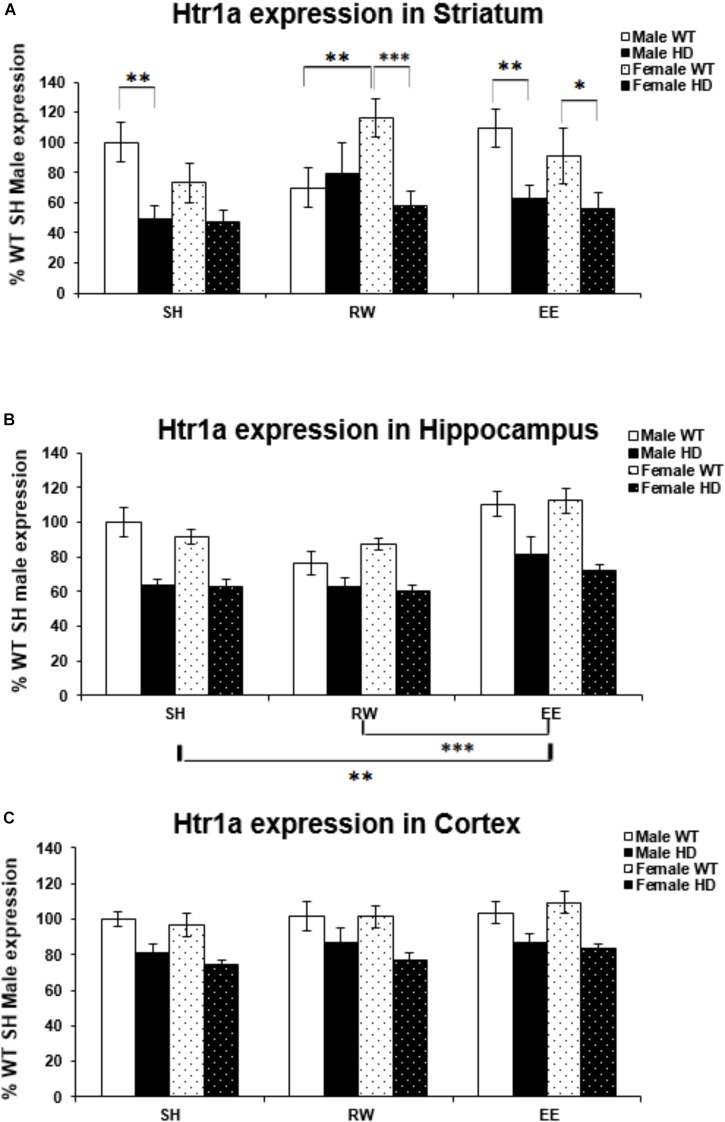
Effects of genotype, environment and sex on Htr1a mRNA levels in 8-week-old WT and HD mice. **(A)** In Striatum, Htr1a expression was decreased significantly in 8-week-old HD mice compared to their WT counterparts (*p* < 0.001). There was a significant interaction between genotype × housing environment × sex (*p* = 0.021). Pairwise comparisons showed significant differences in Htr1a expression between male and female WT mice exposed to wheel running (*p* = 0.009), between female HD and WT exposed to EE (*p* = 0.044) and RW (*p* = 0.001) and between male HD and WT mice exposed to SH (*p* = 0.004) and EE (*p* = 0.008). **(B)** In Hippocampus, Htr1a expression was reduced significantly in 8-week-old HD mice (*p* < 0.001). Housing environment had a significant effect on Htr1a expression (*p* < 0.001). *Post hoc* testing showed that environmental enrichment (*p* = 0.002), but not wheel running (*p* = 0.214), significantly increased levels of Htr1a mRNA. **(C)** In Cortex, Htr1a expression was decreased significantly in 8-week-old HD mice compared to their WT counterparts (*p* < 0.001). Housing environment had no effect on Htr1a expression (*p* = 0.150). *n* = 4–6 per group. Results are represented as mean ± SEM. Pairwise comparisons were conducted using Bonferonni’s adjustment for multiple comparisons: ^∗^*p* < 0.05; ^∗∗^*p* ≤ 0.01; ^∗∗∗^*p* ≤ 0.001.

Huntington’s disease mice had significantly lower levels of Htr1a mRNA than WT mice in the striatum [Figure 2A; *F*(1,70) = 23.938, *p* < 0.001], hippocampus [Figure 2B; *F*(1,72) = 73.098, *p* < 0.001], and cortex [Figure 2C; *F*(1,71) = 39.875, *p* < 0.001]. In the striatum, analysis of Htr1a expression levels revealed a significant interaction between genotype × housing environment × sex [Figure 2A; *F*(2,70) = 4.107, *p* = 0.021]; pairwise comparisons showed that female WT mice exposed to wheel running had significantly higher levels of Htr1a mRNA than their WT male wheel running counterparts (*p* = 0.009). They also demonstrated that female HD mice exposed to EE had significantly less Htr1a mRNA than female WT EE mice (*p* = 0.044) and that female HD mice exposed to RW had significantly less Htr1a mRNA than female WT RW mice (*p* = 0.001). There were also significant differences between male HD and WT mice within the SH (*p* = 0.004) and EE (*p* = 0.008) groups. No change in Htr1a expression in female WT mice exposed to wheel running, in comparison to female WT mice exposed to standard-housing (*p* = 0.061), was evident.

Housing environment significantly affected Htr1a mRNA levels in the hippocampus [Figure 2B; *F*(2,72) = 15.158, *p* < 0.001]; *post hoc* testing demonstrated that EE significantly increased Htr1a expression levels when compared to SH (*p* = 0.002) and RW (*p* < 0.001). In the cortex, there was no effect of housing environment on Htr1a expression levels [Figure 2C; *F*(1,71) = 1.960, *p* = 0.150].

### Region-Specific Changes in Htr1b Gene Expression HD Mice Are Differentially Modulated by Sex and Environment

**FIGURE 3:**
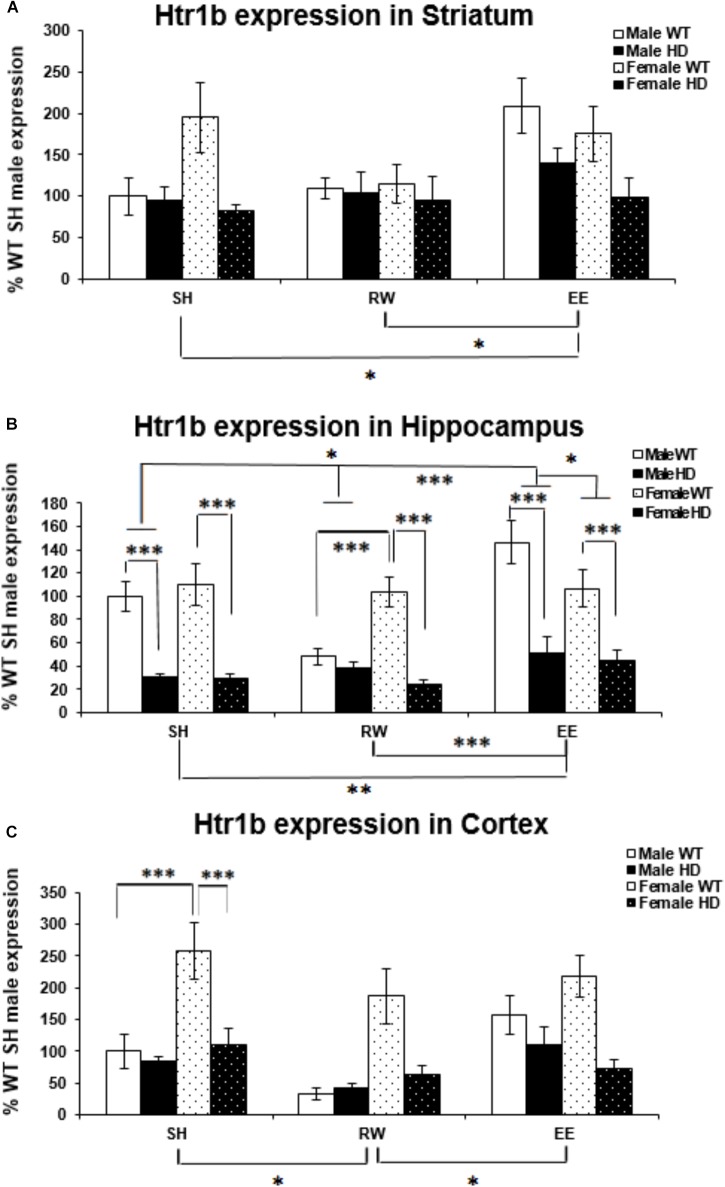
Effects of genotype, environment and sex on Htr1b mRNA levels in 8-week-old WT and HD mice. **(A)** In Striatum, Htr1b expression was significantly affected by genotype in 8-week-old female mice (*p* = 0.002). Housing environment significantly affected Htr1b mRNA levels (*p* = 0.016). *Post hoc* testing showed that environmental enrichment (*p* = 0.046), but not wheel running (*p* = 0.860), significantly changed levels of Htr1b mRNA. There was no significant interaction between housing environment × sex (*p* = 0.063). **(B)** In Hippocampus, Htr1b expression was decreased significantly in 8-week-old HD mice compared to their WT counterparts (*p* < 0.001). Housing environment significantly affected Htr1b mRNA levels (*p* < 0.001). *Post hoc* testing showed that environmental enrichment (*p* = 0.013), but not wheel running (*p* = 0.501), significantly changed levels of Htr1b mRNA. There was a significant interaction between housing environment × sex (*p* = 0.024); pairwise comparisons showed a significant difference between male and female mice held in EE conditions (*p* = 0.040) and no difference between male and female mice held in RW conditions (*p* = 0.069). Male EE mice also showed significantly more Htr1a expression than male SH (*p* = 0.012) and RW (*p* < 0.001) mice. There was a significant interaction between genotype × housing environment × sex (*p* = 0.008); pairwise comparisons showed significant differences in Htr1a expression between male and female WT mice exposed to wheel running (*p* = 0.001) and environmental enrichment (*p* = 0.014). They also showed significant differences between female HD and WT mice raised under SH, RW and EE conditions (*p* < 0.001 for all) and between male HD and WT mice raised under SH and EE conditions (*p* < 0.001 for both). Additionally male WT mice raised in EE conditions had significantly increased levels of Htr1b mRNA when compared to SH (*p* = 0.019) and RW (*p* < 0.001) male WT mice and male WT mice raised in RW conditions had significantly less Htr1b expression when compared to SH (*p* = 0.008) WT male mice. **(C)** In Cortex, Htr1b expression was decreased significantly in 8-week-old female HD mice compared to their WT counterparts (*p* < 0.001). Housing environment significantly affected Htr1b mRNA levels (*p* = 0.005). *Post hoc* testing showed that wheel running (*p* = 0.010), but not environmental enrichment (*p* = 0.921), significantly changed levels of Htr1b mRNA. There was a significant interaction between genotype × sex (*p* < 0.001); pairwise comparisons showed a significant difference between Htr1b expression in male and female WT mice (*p* < 0.001) and a significant difference in Htr1b expression between HD and WT female mice (*p* < 0.001). *n* = 4–6 per group. Results are represented as mean ± SEM. Pairwise comparisons were conducted using Bonferonni’s adjustment for multiple comparisons: ^∗^*p* < 0.05; ^∗∗^*p* ≤ 0.01; ^∗∗∗^*p* ≤ 0.001.)

**FIGURE 4:**
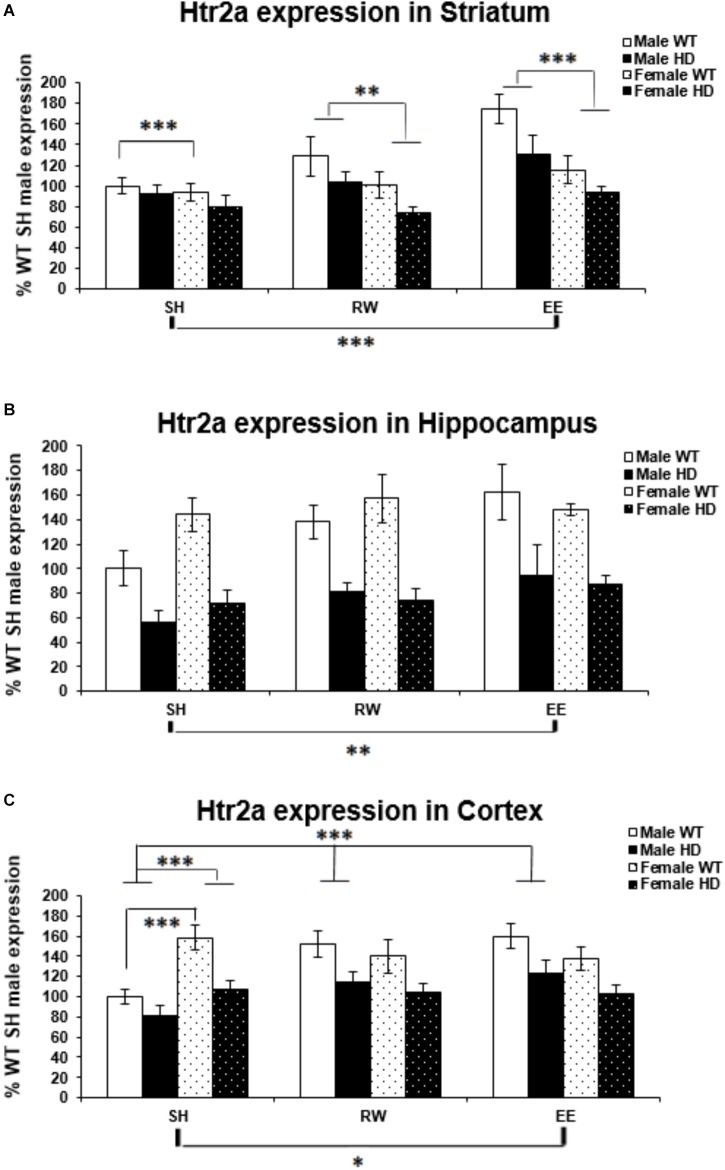
Effects of genotype, environment and sex on Htr2a mRNA levels in 8-week-old WT and HD mice. **(A)** In Striatum, Htr2a expression was reduced significantly in 8-week-old HD mice (*p* = 0.001). Housing environment had a significant effect on Htr2a expression (*p* < 0.001). *Post hoc* testing showed that environmental enrichment (*p* < 0.001), but not wheel running (*p* = 0.461), significantly increased levels of Htr2a mRNA compared to SH. There was also a significant effect of sex on Htr2a expression (*p* < 0.001) and a trend toward an interaction between sex × housing environment (*p* = 0.063); pairwise comparison showed that male mice had significantly higher levels of Htr2a mRNA than their female counterparts when held under EE and RW conditions (*p* < 0.001 and *p* = 0.018, respectively) and that EE significantly increased Htr2a expression levels in male mice compared to SH and RW conditions (*p* < 0.001 and *p* = 0.008, respectively). **(B)** In Hippocampus, Htr2a expression was reduced significantly in 8-week-old HD mice (*p* < 0.001). Housing environment had a significant effect on Htr2a expression (*p* = 0.014). *Post hoc* testing showed that environmental enrichment (*p* = 0.004), but not wheel running (*p* = 0.182), significantly increased levels of Htr2a mRNA. **(C)** In Cortex, Htr2a expression was reduced significantly in 8-week-old HD mice (*p* < 0.001). Housing environment had a significant effect on Htr2a expression (*p* = 0.038). *Post hoc* testing showed that environmental enrichment (*p* = 0.021), but not wheel running (*p* = 0.122), significantly increased levels of Htr2a mRNA. There was a significant interaction between sex × housing environment (*p* < 0.001); pairwise comparison showed that female mice had significantly higher levels of Htr2a expression than their male counterparts when held under SH conditions (*p* < 0.001) and that EE and RW significantly increased Htr2a mRNA levels in male mice compared to SH (*p* < 0.001 and *p* = 0.001, respectively). *n* = 4–6 per group. Results are represented as mean ± SEM. Pairwise comparisons were conducted using Bonferonni’s adjustment for multiple comparisons: ^∗^*p* < 0.05; ^∗∗^*p* ≤ 0.01; ^∗∗∗^*p* ≤ 0.001.)

Htr1b mRNA levels were significantly affected by genotype in the striatum [Figure 3A; *F*(1,61) = 11.221, *p* = 0.002]. Htr1b expression levels were also significantly affected by housing environment [Figure 4A; *F*(2,61) = 4.511, *p* = 0.016]; *post hoc* testing demonstrated that mice exposed to EE conditions had increased Htr1b expression in comparison to SH (*p* = 0.046) and RW (*p* = 0.021) mice.

Htr1b expression levels are reduced in HD hippocampus compared to WT expression levels [Figure 3B; *F*(1,69) = 101.174, *p* < 0.001]. There was also a significant effect of housing environment on Htr1b expression levels in the hippocampus [*F*(2,69) = 9.321, *p* < 0.001]; *post hoc* testing revealed that mice housed in EE conditions had significantly higher levels of Htr1b expression than mice housed in SH conditions (*p* = 0.013) and RW conditions (*p* < 0.001). A significant interaction between housing environment × sex was evident [Figure 3B; *F*(2,69) = 3.981, *p* = 0.024]. Pairwise comparisons showed a significant difference between male and female mice within the EE group (*p* = 0.040), with a trend toward a difference between male and female mice in the RW group (*p* = 0.069); male EE mice also had significantly higher levels of Htr1b expression than male SH (*p* = 0.012) and male RW (*p* < 0.001) mice. A significant interaction between genotype × housing environment × sex was also apparent [Figure 3B; *F*(2,69) = 5.303, *p* = 0.008]; pairwise comparisons showed that female WT RW mice had significantly more Htr1b mRNA than male WT RW mice (*p* = 0.001), while male WT EE mice had significantly more Htr1b mRNA than female WT EE mice (*p* = 0.014). Male WT EE mice demonstrated increased levels of Htr1b expression when compared to male WT SH mice (*p* = 0.019) and male WT RW mice (*p* < 0.001), while male WT RW mice demonstrated significantly decreased levels of Htr1b expression when compared to male WT SH mice (*p* = 0.008). Pairwise comparisons also showed significant differences between HD and WT mice within the female SH (*p* < 0.001), RW (*p* < 0.001) and EE (*p* < 0.001) groups, as well as in the male SH (*p* < 0.001) and male EE (*p* < 0.001) groups, but not within the male RW group (*p* = 0.533).

Htr1b mRNA levels were significantly higher in female mice than in male mice [Figure 3C; *F*(1,55) = 16.284, *p* < 0.001]. They were significantly decreased in the HD cortex [Figure 3C; *F*(1,55) = 24.079, *p* < 0.001]. A significant interaction between genotype × sex was apparent [Figure 3C; *F*(2,55) = 14.911, *p* < 0.001]; pairwise comparisons showed significantly higher levels of Htr1b mRNA in female WT mice when compared to male WT mice (*p* < 0.001), but there was no difference between male and female HD mice (*p* = 0.907). A significant decrease in Htr1b expression was evident in female HD mice when compared to female WT mice (*p* < 0.001), whereas no difference was apparent between male WT and HD mice (*p* = 0.489).

Htr1b expression levels were also significantly affected by housing environment [Figure 3C; *F*(2,55) = 5.901, *p* = 0.005]; *post hoc* testing demonstrated that mice exposed to RW conditions showed decreased Htr1b expression in comparison to SH (*p* = 0.010) and EE (*p* = 0.018) mice.

### Htr2a Gene Expression Is Decreased in HD Mice and Differentially Modulated by Sex and Environment

Expression of the Htr2a gene was decreased in HD mice at 8 weeks of age, when compared to their WT counterparts, in the striatum [Figure 4A; *F*(1,65) = 11.700, *p* = 0.001], hippocampus [Figure 4B; *F*(1,66) = 60.316, *p* < 0.001], and cortex [Figure 4C; *F*(1,67) = 30.580, *p* < 0.001].

In the striatum a significant effect of sex [Figure 4A; *F*(1,65) = 18.304, *p* < 0.001] indicated a significantly higher level of Htr2a mRNA in male mice then in female mice. There was a trend of interaction between sex and housing environment [Figure 4A; *F*(2,65) = 2.915, *p* = 0.063]; pairwise comparisons indicated that there was an increase in Htr2a expression in male mice, when compared to female mice, within the EE (*p* < 0.001) and wheel running (*p* = 0.018) groups, but not within the SH group (*p* = 0.452). And also that EE increased Htr2a expression compared to standard housing (*p* < 0.001).

Housing in EE condition increased Htr2a expression in the hippocampus [Figure 4B; *F*(2,66) = 4.592, *p* = 0.014, SH vs EE; *p* = 0.004, SH vs RW; *p* = 0.182]. In the cortex, a significant interaction between sex and housing environment [Figure 4C; *F*(2,67) = 9.606, *p* < 0.001], followed by pairwise comparison, indicated that female SH mice had significantly higher levels of Htr2a mRNA than their male SH littermates (*p* < 0.001); In male mice, EE (*p* < 0.001) and wheel running (*p* = 0.001) conditions increased Htr2a expression in comparison to SH conditions.

### Region-Specific Htr2c Gene Expression Changes in HD Mice Are Differentially Modulated by Sex and Environment

**FIGURE 5:**
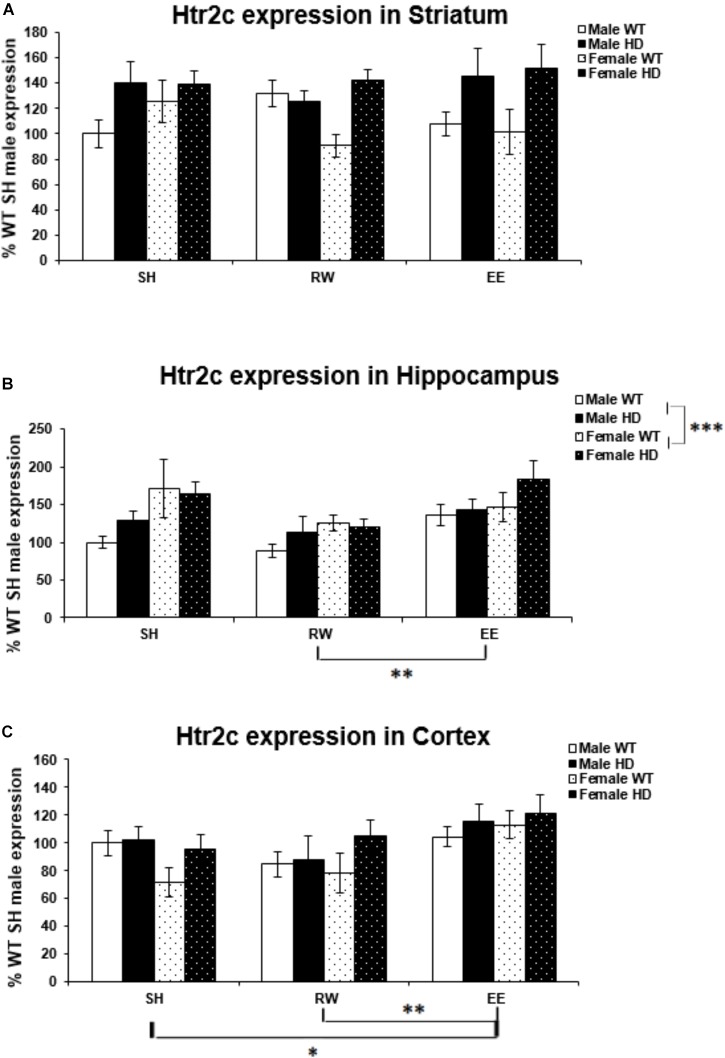
Effects of genotype, environment and sex on Htr2c mRNA levels in 8-week-old WT and HD mice. **(A)** In Striatum, Htr2c expression was increased significantly in 8-week-old HD mice (*p* < 0.001). Housing environment had no effect on Htr2c expression (*p* = 0.912). **(B)** In Hippocampus, Genotype did not significantly affect Htr2c expression in 8-week-old HD mice (*p* = 0.149). Housing environment had a significant effect on Htr2c expression (*p* = 0.004). *Post hoc* testing showed that wheel running mice had significantly less Htr2c mRNA than mice exposed to EE (*p* = 0.003), and showed a trend toward less Htr2c mRNA than SH mice (*p* = 0.054). Male mice demonstrated significantly less Htr2c mRNA than female mice (*p* = 0.001). **(C)** In Cortex, HD mice demonstrated no change in levels of Htr2c mRNA compared to WT mice (*p* = 0.059). Housing environment had a significant effect on Htr2c expression (*p* = 0.006). *Post hoc* testing showed that environmental enrichment (*p* = 0.040), but not wheel running (*p* = 0.846), significantly increased levels of Htr2c mRNA. *n* = 4–6 per group. Results are represented as mean ± SEM. Pairwise comparisons were conducted using Bonferonni’s adjustment for multiple comparisons: ^∗^*p* < 0.05; ^∗∗^*p* ≤ 0.01; ^∗∗∗^*p* ≤ 0.001.

There was a significant difference in Htr2c expression levels between WT and Huntington’s disease mice in the striatum [Figure 5A; *F*(1,71) = 15.306, *p* < 0.001]. There was no difference in Htr2c expression between HD and WT mice in the hippocampus [Figure 5B; *F*(1,70) = 2.134, *p* = 0.149] and likewise in the cortex [Figure 5C; *F*(1,71) = 3.712, *p* = 0.059].

Female mice had significantly higher levels of Htr2c expression in the hippocampus than their male counterparts [Figure 5B; *F*(1,70) = 11.543, *p* = 0.001]. However, there was no difference in expression levels between the sexes in the striatum [Figure 5A; *F*(1,71) = 0.000, *p* = 0.997], or cortex [Figure 5C; *F*(1,71) = 0.075, *p* = 0.785].

Housing environment had no effect on Htr2c expression in the striatum [Figure 5A; *F*(1,71) = 0.093, *p* = 0.912]. In the hippocampus housing environment had a significant effect on Htr2c mRNA levels [Figure 5B; *F*(2,70) = 6.166, *p* = 0.004]; *post hoc* testing demonstrated that RW mice have significantly lower levels of Htr2c mRNA than EE mice (*p* = 0.003). There was also a trend toward lower levels of Htr2c mRNA in RW mice when compared to SH mice (*p* = 0.054). In the cortex there was again a significant effect of housing environment [Figure 5C; *F*(1,71) = 5.541, *p* = 0.006]; *post hoc* testing demonstrated that EE mice have significantly higher levels of Htr2c mRNA than SH mice (*p* = 0.040) and RW mice (*p* = 0.009).

### Behavioral Pharmacological Analysis of 5-HT_2_ Receptors Shows 2 Weeks of EE Boosts the Behavioral Response to Agonism of These Receptors in 8-Week-Old HD Mice

**FIGURE 6:**
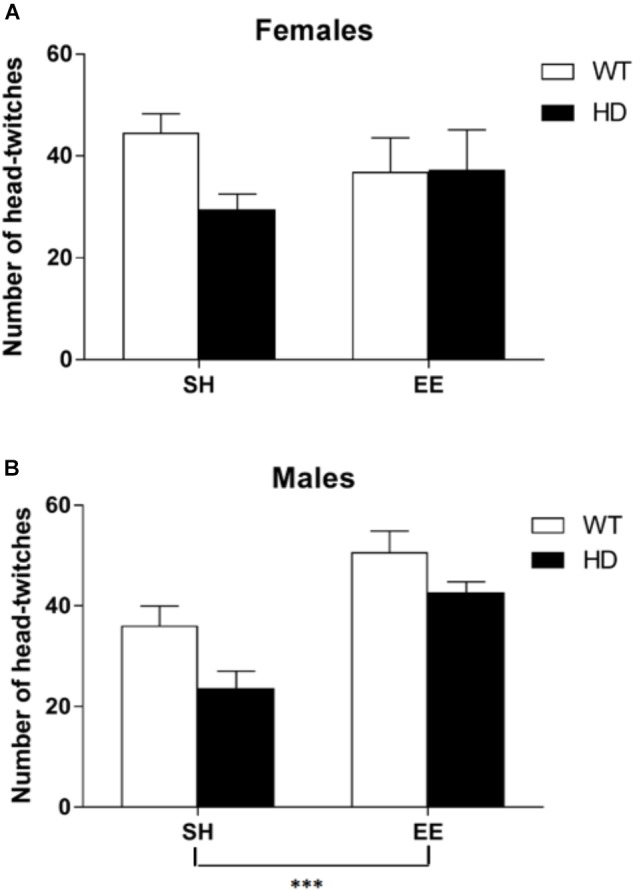
Environmental enrichment induces a sexually dimorphic increase in the number of DOI-induced head-twitches in wild-type and HD mice at 8 weeks of age. The number of head-twitches induced by acute administration of the 5-HT_2_ receptor agonist (DOI) was reduced in HD mice when compared to their wild-type counterparts (*p* = 0.008). There was no overall difference between male and female mice in number of head-twitches. **(A)** There was no significant effect of housing conditions in female mice; *n* = 7–10. **(B)** However, there was a significant effect of housing condition (*p* = 0.010) and a significant interaction between sex and housing condition (*p* = 0.012) with *post hoc* testing showing that environmental enrichment significantly increased the number of head-twitches in both wild-type and HD male mice; *n* = 6–11 (*p* < 0.001). Results are represented as mean ± SEM ^∗∗∗^*p* < 0.001.

The administration of a 5-HT_2A/2C_ receptor agonist, (±)DOI, induced head-twitches in WT and HD mice and this effect was reduced in both female and male HD mice [Figure 6, main effects of genotype, *F*(1,70) = 7.6, *p* = 0.008, and housing condition *F*(1,70) = 7.0, *p* = 0.010, and a significant interaction between sex and housing condition, *F*(1,70) = 6.7, *p* = 0.012]. *Post hoc* testing showed that EE significantly increased the number of head-twitches in both WT and HD male mice (*p* < 0.001) but not in female mice of either genotype (Figure 6).

## Discussion

In this study, we have discovered various significant effects on gene expression due to genotype, environment, sex and brain region. This provides new insight into the pathogenesis of HD, and the therapeutic impacts of EE and exercise on the cognitive, psychiatric and motor symptoms of this currently incurable disease. Furthermore, the sexually dimorphic and region-specific effects we discovered may provide new insight into depression in HD (where sexual dimorphism occurs) and identify brain regions and molecular pathways that can be targeted with future therapeutic approaches.

The most intriguing change in gene expression after exposure to voluntary wheel running was an increase in SerT mRNA levels in the hippocampus in all four groups. An increase in serotonin transporter levels could indicate an increased ability to reuptake and recycle serotonin from the synaptic cleft in the hippocampus. SerT mRNA levels are reduced in the rat raphe nuclei after 3 weeks of running (Greenwood et al., 2005) and in a future experiment it would be interesting to investigate whether this is the case in HD mice.

There were significant decreases in Htr1a mRNA levels in 8-week-old male and female R6/1 HD mice in the hippocampus, cortex and striatum. Similar reduction in the hippocampus and cortex of 12-week-old male and female R6/1 HD mice have been demonstrated previously (Pang et al., 2009). This suggests that the transcriptional down-regulation of Htr1a occurs early in pathogenesis, prior to onset of behavioral deficits. Furthermore, our prior evidence of abnormal signaling through 5-HT1a autoreceptors in the raphe (Renoir et al., 2013) are consistent with transcriptional dysregulation of the Htr1a gene, and associated functional consequences.

The current study illustrates that EE and voluntary wheel-running paradigms do not have the same effect on gene expression patterns, far from it. In particular, Htr1b expression in the hippocampus and cortex of male WT mice is decreased by voluntary wheel running but increased by EE. In the cortex the same effect is apparent in the male R6/1 HD mice with a significant decrease in Htr1b mRNA levels due to voluntary wheel running and a significant increase caused by exposure to EE.

This study demonstrated dysregulation of the serotonergic system in the striatum, cortex and hippocampus of R6/1 HD mice, at early stages of the disease process. The R6/1 HD mouse model does not typically show any motor symptoms before 12 weeks of age (Pang et al., 2006; Nithianantharajah et al., 2008), however, both the male and female mice start to show cognitive behavioral changes (Mazarakis et al., 2005; Nithianantharajah et al., 2008; Mo et al., 2013, 2014a, 2015) including specific deficits in hippocampal-dependent, but not hippocampal-independent memory (Nithianantharajah et al., 2008; Mo et al., 2014b). Therefore, any molecular and cellular changes seen in both male and female HD mice at 8 weeks of age in the present study are more likely to be involved in the onset of these cognitive symptoms.

Previous findings from our laboratory showed that female, but not male, R6/1 HD mice exhibited a depressive-like phenotype at 8–12 weeks of age (Pang et al., 2009; Renoir et al., 2011). In the present study, while Htr2c expression levels in 8-week-old female HD mice did not differ from those seen in WT in any of the regions examined, male HD mice had significantly increased Htr2c expression in the striatum. This specific increase in males may reflect a potential compensatory mechanism. With respect to potential mechanisms mediating this sexual dimorphism, sex hormones are the most obvious candidate. We have previously demonstrated abnormalities of the hypothalamic-pituitary-gonadal (HPG) axis in these HD mice (Du et al., 2015), and this may contribute to our observed findings.

Our findings in the present study provide new evidence that 2 weeks of EE can correct certain deficits in gene expression in R6/1 HD mice. The effect was restricted to male R6/1 HD mice, with no effect of EE on female R6/1 HD gene expression. The differential effect of EE on gene expression in male and female WT mice has not been previously demonstrated. We have found that EE can produce changes in gene expression (in a previous study focusing on BDNF) of a large magnitude in the brain of male mice where none occurs in females (Zajac et al., 2010).

Levels of Htr2a mRNA in 8-week-old male HD mice exposed to 2 weeks of EE were comparable to, or exceeded, levels in WT male SH mice, showing a rescue of the deficit caused by the HD transgene. These results differ from those in 12-week-old HD mice showing no change in Htr2a expression 4 weeks of EE (Pang et al., 2009). This agrees with the hypothesis that the time period of the EE paradigm is critical with regard to cellular responses associated with transcriptional regulation. This hypothesis is also relevant to exercise interventions and could also explain the difference between the present study and our previous study in which a wheel-running intervention occurred between 8 and 12 weeks of age (Renoir et al., 2012). Changes in gene expression in response to a novel environment are likely to be transient, with gene expression returning after a period of time to steady-state levels even if the environmental changes persist. Transient changes in the effects of EE are biologically relevant as they could affect long-lasting structural changes within cells.

We therefore used a test of 5-HT_2A/2C_ receptor function to assess whether EE-mediated change in Htr2a receptor expression was translated into a functional increase in receptor activity. The study showed that exposure to 2 weeks of EE increased the number of head-twitches seen after an injection of the 5-HT2 receptor agonist, DOI, in both HD and WT male mice, suggesting that EE does indeed increase the functional activity of 5-HT_2A_ receptors in the mouse brain.

One limitation of the present study is that our gene expression measures were performed only at the mRNA level. It will be of interest in future studies to assess the impacts of these environmental interventions, as well as the associated effects of genotype, sex and brain region, at the protein level, using complementary approaches such as Western analysis and radioligand autoradiography, which would also provide more spatial resolution.

In this study, we have demonstrated a functional effect of early EE on behavioral pharmacology in WT mice, as well as a mouse model of a neurodegenerative disease. Our results provide new insight into the underlying mechanisms mediating the beneficial effects of EE, and will inform the development of this approach, and associated molecular targets, for future therapeutic interventions.

## Author Contributions

MZ and AH designed the experiments. MZ and TR performed the experiments. VP, SL, WA, and MvdB provided technical advice and support in preparing the manuscript and figures. MZ, TR, and AH wrote the manuscript. AH provided the funding for the experiments.

## Conflict of Interest Statement

The authors declare that the research was conducted in the absence of any commercial or financial relationships that could be construed as a potential conflict of interest.
